# Characteristics of SARS-CoV-2 Reinfection and Ancestral RBD-Blocking Antibody Levels: A Cross-Sectional Study in the Post-Zero-COVID Era from Shanghai

**DOI:** 10.3390/vaccines14060520

**Published:** 2026-06-10

**Authors:** Chen Chen, Yuanfei Zhu, Huiting Wang, Fei Wu, Youhua Xie, Qingqing Jia, Yang Yang, Jiangjiang Lyu, Junqiang Qu, Qiao Wang, Fan Wu

**Affiliations:** 1 Shanghai Institute of Infectious Disease and Biosecurity, Fudan University, Shanghai 200032, China; chenchen21@m.fudan.edu.cn (C.C.); zhuyuanfei@fudan.edu.cn (Y.Z.); fwu22@m.fudan.edu.cn (F.W.); yhxie@fudan.edu.cn (Y.X.); 23111020015@m.fudan.edu.cn (Q.J.); 2 Key Laboratory of Medical Molecular Virology (MOE/NHC/CAMS), Shanghai Frontiers Science Center of Pathogenic Microorganisms and Infection, School of Basic Medical Sciences, Fudan University, Shanghai 200032, China; 20111010067@fudan.edu.cn; 3 Minhang District Center for Disease Control and Prevention, Shanghai 201101, China; 22211020023@m.fudan.edu.cn; 4 Xietu Street Community Health Service Center, Xuhui District, Shanghai 200032, China; 5 Longhua Street Community Health Service Center, Xuhui District, Shanghai 200232, China; lhfbkqjq@163.com; 6 Shanghai Fifth People’s Hospital, Fudan University, Shanghai 200240, China; 7 Shanghai Medical College, Fudan University, Shanghai 200032, China

**Keywords:** COVID-19, Omicron variant, SARS-CoV-2 reinfection, epidemiological characteristics, vaccine

## Abstract

**Background**: SARS-CoV-2 reinfections increased substantially after the emergence of Omicron variants. **Methods**: We conducted a cross-sectional study of 2095 individuals with prior Omicron BA.2 infection in Shanghai, China, during the early post-zero-COVID period. Data on demographics, infection history, and lifestyle factors were collected via questionnaire, and blood samples were obtained for ancestral RBD-blocking antibody measurement. **Results**: Meeting WHO physical activity recommendations (≥600 MET-min/week) was associated with lower reinfection odds (OR = 0.59, 95% CI: 0.46–0.74, *p* < 0.001). The overall median ancestral RBD-blocking antibody level was 263.93 U/mL (IQR: 36.41–331.87). Older age was associated with lower ancestral RBD-blocking antibody levels (β = –0.0038 per year, 95% bootstrap CI: –0.0057 to –0.0019, *p* < 0.001). All vaccinated groups had significantly higher ancestral RBD-blocking antibody levels than unvaccinated individuals: partially vaccinated (β = 0.4440, 95% CI: 0.1569 to 0.6830, *p* < 0.001), fully vaccinated (β = 0.8516, 95% CI: 0.7464 to 0.9595, *p* < 0.001), homologous booster (β = 1.0297, 95% CI: 0.9408 to 1.1223, *p* < 0.001), and heterologous booster (β = 1.0838, 95% CI: 0.9387 to 1.2226, *p* < 0.001). Time since last immune event was inversely associated with ancestral RBD-blocking antibody levels (β = –0.0232 per month, 95% CI: –0.0385 to –0.0077, *p* = 0.0031). **Conclusions**: In this cross-sectional study, meeting WHO physical activity recommendations was associated with 41% lower odds of SARS-CoV-2 reinfection, although reverse causality cannot be ruled out. All vaccinated groups had higher ancestral RBD-blocking antibody levels than unvaccinated individuals. Older age and longer time since last immune event were associated with lower ancestral RBD-blocking antibody levels. These associations need confirmation in prospective, well-powered studies.

## 1. Introduction

With the emergence of Omicron (B.1.1.529) variant and its sublineages [1,2,3], severe acute respiratory syndrome coronavirus 2 (SARS-CoV-2) reinfections have markedly increased—likely due to their enhanced transmissibility and immune evasion compared with earlier variants [4,5,6,7]. Moreover, past infection provides only 45.3% protection against Omicron reinfection, which wanes more rapidly than against pre-Omicron variants [8]. Previous studies have also suggested that SARS-CoV-2 reinfection is associated with significant health risks, including increased risk of long-term sequelae, multi-organ damage, and accelerated aging processes [9,10,11]. Therefore, further studies are needed to elucidate the epidemiological characteristics, risk factors, and humoral immune response associated with SARS-CoV-2 reinfection. These insights are essential for informing evidence-based interventions to mitigate viral transmission.

Shanghai, the largest city in eastern China, experienced its first COVID-19 wave caused by the Omicron BA.2 variant between March and June 2022 [12,13,14]. Prior to December 2022, China maintained a “dynamic zero-COVID” policy aimed at eliminating local transmission of SARS-CoV-2 [15,16,17]. Under this policy, routine nucleic acid amplification testing (NAAT) was extensively implemented to enable highly sensitive surveillance for detecting SARS-CoV-2 infection. Following the policy transition in late 2022, China experienced a nationwide Omicron BA.5 wave, resulting in widespread and rapid population exposure to this variant [18,19]. Given that Shanghai experienced two consecutive Omicron waves (BA.2 and BA.5) with a distinct infection background, this raises questions about the incidence and determinants of Omicron reinfection during the post-zero-COVID era. Therefore, we conducted a cross-sectional study in Shanghai between 6 January and 7 April 2023. The objectives of this study were to characterize the epidemiological features of Omicron reinfection, to compare the clinical presentations of reinfection with those of primary infection, and to assess population ancestral receptor-binding domain (RBD)-blocking antibody levels and their associated factors. This study provides empirical data from the post-zero-COVID period in Shanghai, China, and contributes to the global understanding of reinfection characteristics in the Omicron era.

## 2. Materials and Methods

### 2.1. Study Design

A community-based cross-sectional survey was conducted in Xuhui District, Shanghai between 6 January and 7 April 2023, among individuals with a documented primary SARS-CoV-2 infection during the Omicron BA.2 outbreak (March–June 2022). Eligible participants were identified through the Shanghai Municipal Infectious Disease Surveillance Information System [14,20]. Inclusion criteria were provision of informed consent and documented primary infection between March and June 2022. Exclusion criteria were any documented SARS-CoV-2 infection prior to March 2022 or refusal to participate. A total of 3863 eligible individuals were identified from the surveillance system. The sampling method was convenience sampling: all eligible individuals were invited, and all who gave informed consent were enrolled. Trained research staff administered a structured questionnaire and collected peripheral blood samples for ancestral RBD-blocking antibody testing.

The minimum sample size was calculated using the formula commonly used in cross-sectional studies: $n=\frac{Z_{1-\alpha /2}^{2}p\left(1-p\right)}{d^{2}}$. Given a two-sided significance level of 5%, an anticipated reinfection rate of 18% based on a previous study [21], a 3% allowable margin of error, and considering a potential non-response rate of 20%, the required sample size was 788.

The study was approved by the Ethics Committee of Fudan University (Ethical Approval Numbers: 2022-12-1022). Written informed consent was obtained from all participants and the data collected from the study was anonymized for analysis.

### 2.2. Definition of SARS-CoV-2 Reinfection

Following the adjustment of the “dynamic zero-COVID” policy in December 2022, routine large-scale nucleic acid amplification testing was largely discontinued. In this study, among participants with documented primary infection during March–June 2022, SARS-CoV-2 reinfection was defined as any infection after 1 December 2022, meeting the following criteria: Confirmed reinfection was defined as participants who tested positive for SARS-CoV-2 nucleic acid or antigen (by self-report or laboratory record review), regardless of symptoms. Probable reinfection was defined as participants without laboratory confirmation who had an acute onset or worsening of any COVID-19-related symptoms (fever, fatigue, cough, sore throat, hyposmia/anosmia, nasal congestion, runny nose, myalgia, conjunctivitis, diarrhea, or others) and an epidemiological history (close contact with a confirmed case or a person with similar symptoms). The reinfection proportion was calculated as the sum of confirmed and probable reinfections divided by the total number of participants.

### 2.3. Data Collection

The structured questionnaire covered five aspects: (1) Basic demographic information included age, sex, height, weight and education level. (2) Previous medical history included comorbidities and allergies. (3) Lifestyle factors included alcohol consumption, smoking, and total physical activity per week. Physical activity was assessed using the Global Physical Activity Questionnaire (GPAQ) [22], which records metabolic equivalents (MET-min/week) from occupational, transport, household, and leisure-time activities. The WHO recommends a minimum total physical activity level of 600 MET-min/week. (4) COVID-19 infection history included infection status, date of infection, diagnostic method, and symptoms. (5) COVID-19 vaccination status. Vaccination data were obtained from the National Immunization Program Information System. For the reinfection analysis, vaccination status was assessed as of 1 December 2022 (the start of the reinfection risk window), based on doses received on or before that date. For the ancestral RBD-blocking antibody analysis, vaccination status was assessed as of the date of blood collection (January–April 2023). In both analyses, participants were categorized into five groups: unvaccinated (no dose); partially vaccinated (primary immunization series incomplete, or completed less than 14 days before the respective index date); fully vaccinated (primary immunization completed at least 14 days before the index date, i.e., 1 dose of adenovirus vector vaccine, 2 doses of inactivated vaccine, or 3 doses of recombinant subunit vaccine); homologous booster vaccinated (fully vaccinated and received an additional dose of the same vaccine platform at least 14 days before the index date); and heterologous booster vaccinated (fully vaccinated and received an additional dose of a different vaccine platform at least 14 days before the index date).

### 2.4. Ancestral RBD-Blocking Antibody Detection

Plasma ancestral RBD-blocking antibody levels were quantified using the cPass SARS-CoV-2 Neutralization Antibody Detection Kit (GenScript Biotech, Nanjing, China) according to the manufacturer’s protocol. Briefly, 20 μL of plasma sample, positive and negative controls were diluted 1:9 in buffer, mixed with an equal volume of HRP-RBD solution, and incubated at 37 °C for 30 min. The mixture was added to a capture plate and incubated for an additional 15 min at 37 °C. After washing four times with diluted wash buffer, 100 μL of TMB substrate was added to each well, followed by incubation in the dark at room temperature for 15 min. The enzymatic reaction was stopped with 50 μL stop solution. The absorbance (OD 450 nm) was measured using a microplate reader. Plasma ancestral RBD-blocking antibody levels were expressed as percentage signal inhibition calculated from OD values. Samples with ≥30% inhibition were considered positive for ancestral RBD-blocking antibody [23]. Percentage inhibition was converted to antibody titers (U/mL) using a WHO International Standard conversion tool [24].

$$\%Signal\;inhibition=\left(1-\frac{OD\;value\;of\;serum\;sample}{OD\;value\;of\;negative\;control}\right)\times 100\%$$

The cPass assay measures the ability of antibodies to block the interaction between the ancestral (wild-type) SARS-CoV-2 RBD and ACE2. Therefore, the measured levels primarily reflect antibody-mediated blocking of the ancestral SARS-CoV-2 RBD–ACE2 interaction and may not fully capture functional neutralization, particularly against Omicron subvariants. Throughout this manuscript, we refer to these measurements as “ancestral-strain RBD-blocking antibodies” or “ancestral RBD-blocking antibodies”, with the understanding that they are not Omicron-specific.

### 2.5. Statistical Analysis

Categorical variables were presented as frequencies (percentages), and between-group differences were analyzed using chi-square test or Fisher’s exact test, as appropriate. Normally distributed continuous variables were expressed as mean ± standard deviation (SD) and compared using Student’s *t* test, while non-normal distributed continuous variables were expressed as median (interquartile range, IQR) and compared using Mann–Whitney U test. The McNemar test was used for paired comparisons of categorical variables.

A multivariable logistic regression analysis was conducted to identify factors associated with SARS-CoV-2 reinfection, estimating odds ratios (ORs) with their 95% confidence intervals (CIs). Covariates were selected based on a review of the literature and clinical knowledge, considering variables known or suspected to be associated with reinfection. The final multivariable logistic regression model included age, sex, BMI, education level, comorbidities, smoking, alcohol consumption, physical activity, primary infection presentation, viral shedding duration of primary infection, vaccination status, and post-primary-infection vaccination.

To account for waning immunity, we calculated a continuous variable “time since last immune event” (in months) as the interval between the date of blood collection and the latest of the following dates (whichever occurred last): (1) date of primary infection (always available), (2) date of the latest vaccine dose (if any), and (3) date of reinfection (if any). This rule ensures that the variable reflects the latest immunologically relevant event for every participant.

A left-censored Tobit regression with bootstrap standard errors (1000 resamples) was used to identify factors associated with ancestral RBD-blocking antibody levels, as 21.4% of participants had titers below the limit of detection (LOD = 16.5 U/mL). The dependent variable was log_10_-transformed antibody titers, with left censoring at log_10_ (LOD) = 1.217. Independent variables included age (continuous), sex, BMI (continuous), education level, comorbidity, smoking, alcohol consumption, physical activity, reinfection status, vaccination status, and time since last immune event (months). As a sensitivity analysis, we also performed quantile regression at the median (τ = 0.5) using the same set of covariates.

Because multiple comparisons were performed, *p*-values should be interpreted descriptively. No adjustment for multiple testing was applied, as the analyses were primarily exploratory. Findings with borderline significance should be interpreted with caution.

All analyses were performed using R version 4.4.1 (R Foundation for Statistical Computing, Vienna, Austria), and a two-sided *p* < 0.05 was considered statistically significant.

## 3. Results

### 3.1. Baseline Characteristics of the Study Participants

**Figure A1:**
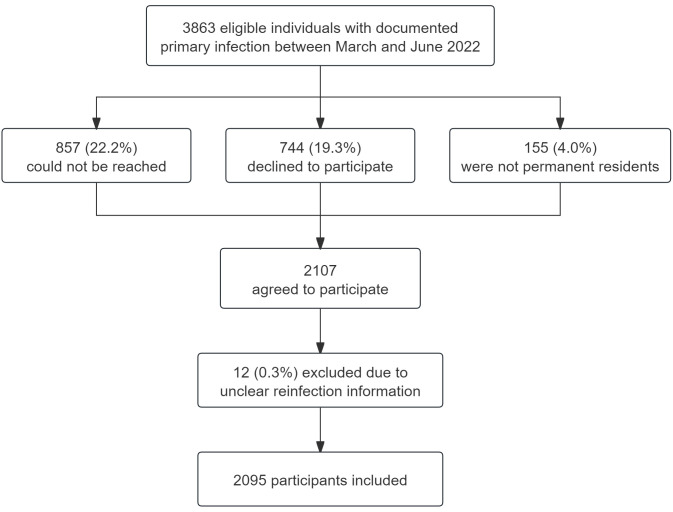
Recruitment flow diagram.

Among 3863 eligible individuals with documented primary Omicron BA.2 infection between March and June 2022, 857 (22.2%) could not be reached due to invalid or missing contact information, 744 (19.3%) declined to participate, and 155 (4.0%) were not permanent residents of Shanghai. The remaining 2107 agreed to participate, of whom 12 were excluded because of unclear reinfection information, yielding a final analytical sample of 2095 participants (Figure A1). We compared participants (*n* = 2095) with non-participants (*n* = 1768) with respect to age and sex. As shown in Table A1, participants were significantly older than non-participants (median: 56 years, IQR: 42–68 vs. 52 years, IQR: 33–66; *p* < 0.001). No statistically significant difference was observed for sex (male: 46.0% [963/2095] vs. 46.7% [826/1768], *p* = 0.650).

Among the 2095 participants, 465 met the criteria for SARS-CoV-2 reinfection, comprising 357 (76.8%) confirmed cases and 108 (23.2%) probable cases. During the observation period from 1 December 2022 to 7 April 2023, the overall incidence of SARS-CoV-2 reinfection was 22.2% (465/2095). After direct standardization to the age-sex distribution of the Shanghai permanent resident population [25], the standardized reinfection rate was 21.8%.

Baseline characteristics of participants stratified by reinfection status are shown in Table 1. In the non-reinfection group, the median age was 56 years (IQR: 42–68), and 46.69% (761/1630) were male. In the reinfection group, the median age was 57 years (IQR: 42–68), and 43.44% (202/465) were male. No statistically significant differences were observed between the two groups with respect to age, sex, body mass index (BMI), education level, comorbidities, allergies, smoking status, or alcohol consumption (*p* > 0.05 for all comparisons). However, the proportion of participants meeting the WHO-recommended physical activity level (≥600 MET-min/week) was significantly higher in the non-reinfection group than in the reinfection group (40.06% [653/1630] vs. 29.46% [137/465], *p* < 0.001).

Regarding the primary infection episode, asymptomatic presentation was significantly more frequent in the non-reinfection group than in the reinfection group (41.04% [669/1630] vs. 35.27% [164/465], *p* = 0.025), and a longer viral shedding duration (>14 days) was also more common in the non-reinfection group (14.72% [240/1630] vs. 10.75% [50/465], *p* = 0.029).

With respect to vaccination status, a significant difference in the overall distribution of vaccination categories was observed between the two groups (*p* = 0.013). Specifically, the non-reinfection group had slightly higher proportions of individuals who received only a primary vaccination series (20.14% [328/1630] vs. 19.57% [91/465]) or a heterologous booster (4.91% [80/1630] vs. 1.29% [6/465]). Furthermore, the proportion of participants who received vaccination after their primary infection was significantly higher in the non-reinfection group compared with the reinfection group (9.58% [156/1630] vs. 5.38% [25/465], *p* = 0.004).

### 3.2. Factors Associated with SARS-CoV-2 Reinfection

Univariable and multivariable logistic regression analyses for SARS-CoV-2 reinfection are presented in Table 2. In the univariable analysis, total physical activity per week, primary infection presentation, viral shedding of primary infection, vaccination status, and vaccination after primary infection were significantly associated with SARS-CoV-2 reinfection (all *p* < 0.05).

Multivariable logistic regression analysis revealed that symptomatic primary infection was significantly associated with increased reinfection odds (OR = 1.37, 95% CI: 1.09–1.72, *p* = 0.008). In contrast, meeting WHO physical activity recommendations (≥600 MET-min/week) (OR = 0.59, 95% CI: 0.46–0.74, *p* < 0.001) and heterologous booster vaccination (OR = 0.31, 95% CI: 0.11–0.89, *p* = 0.029) were significantly associated with lower reinfection odds. However, the estimate for heterologous booster vaccination was unstable and should be interpreted with caution, as it was based on a small subgroup (86 participants) with only six reinfection events. Notably, vaccination after primary infection was associated with lower reinfection odds in univariable analysis (OR = 0.54, 95% CI: 0.35–0.83, *p* = 0.005). However, this association became non-significant in the multivariable model (OR = 0.84, 95% CI: 0.48–1.43, *p* = 0.505), suggesting that the univariable association was largely explained by other covariates.

The final multivariable model had an events-per-variable ratio of 17.9. The Hosmer–Lemeshow test indicated good calibration (χ^2^ = 5.613, df = 8, *p* = 0.691). The area under the ROC curve (AUC) was 0.62 (95% CI: 0.60–0.65), indicating modest discrimination. Variance inflation factors for all covariates were below 2.0, confirming no problematic multicollinearity.

To address potential misclassification of probable reinfections, we repeated the multivariable logistic regression analysis including only confirmed reinfections (n = 357) and non-reinfected participants (n = 1630). As shown in Table A2, meeting WHO physical activity recommendations remained significantly associated with lower reinfection odds (OR = 0.61, 95% CI: 0.47–0.79, *p* < 0.001). However, the associations for symptomatic primary infection (OR = 1.22, 95% CI: 0.95–1.57, *p* = 0.127) and heterologous booster vaccination (OR = 0.32, 95% CI: 0.10–1.01, *p* = 0.052) were no longer statistically significant. For symptomatic primary infection, the association observed in the primary analysis was not robust to the exclusion of probable reinfections. For heterologous booster vaccination, the analysis was limited by the very small number of events (only 5 confirmed reinfection events among 85 recipients), rendering the estimate imprecise. Both findings should therefore be considered inconclusive, pending validation in larger studies. In this sensitivity analysis, female sex (OR = 1.34, 95% CI: 1.02–1.75, *p* = 0.038) and current smoking (OR = 1.62, 95% CI: 1.06–2.47, *p* = 0.026) were associated with higher reinfection odds, but these findings also require replication in larger studies.

### 3.3. Comparison of Clinical Symptoms Between Primary Infection and Reinfection

**Figure 1:**
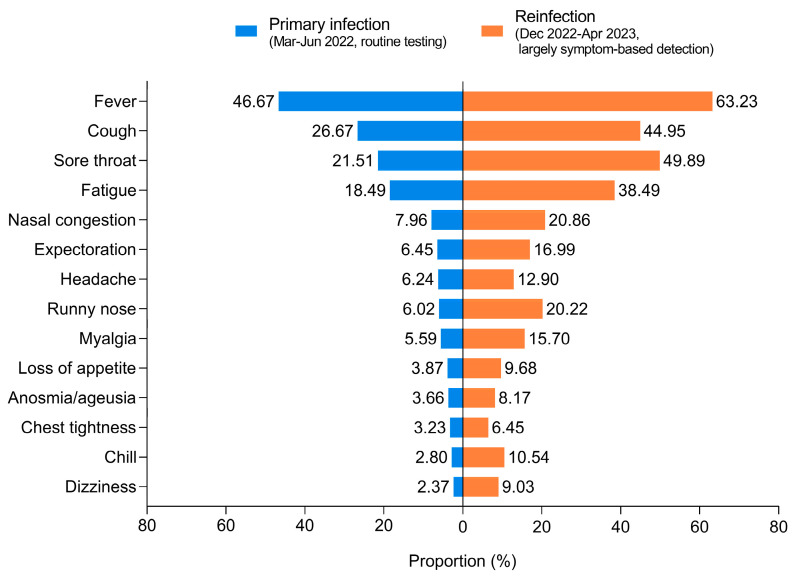
Back-to-back bar chart of symptoms distribution between primary infection and reinfection. Bars to the left (blue) represent symptom proportions during primary infection, and bars to the right (orange) represent proportions during reinfection. Symptom comparisons are confounded by surveillance bias: primary infection was detected under routine nucleic acid amplification testing, while reinfections were largely symptom-triggered after testing was discontinued.

Among the 465 participants with SARS-CoV-2 reinfection, the proportion of asymptomatic infection during the primary infection was higher than that during reinfection (35.27% vs. 7.10%) (Table A3). Regarding the symptomatic cases, the five most common symptoms were fever, cough, sore throat, fatigue, and nasal congestion, which were similar between both infections (Figure 1). The reported prevalence of most individual symptoms was higher during reinfection than during primary infection.

These comparisons should be interpreted with caution, as they are subject to surveillance bias. During the primary infection (March–June 2022), routine nucleic acid amplification testing was in place, enabling the detection of asymptomatic cases. In contrast, reinfections occurred after December 2022, following the adjustment of the zero-COVID policy, when large-scale testing was largely discontinued. Thus, reinfections were predominantly identified when symptoms prompted testing, leading to under-ascertainment of asymptomatic reinfections. Consequently, the observed higher symptom burden during reinfection likely reflects surveillance bias rather than a true increase in clinical severity.

### 3.4. Ancestral RBD-Blocking Antibody Levels in the Study Population

Among the 2095 participants, the median ancestral RBD-blocking antibody level was 263.93 U/mL (IQR: 36.41–331.87). Distributions of ancestral RBD-blocking antibody levels by participant characteristics are shown in Table 3.

Ancestral RBD-blocking antibody levels differed significantly by age group (*p* < 0.001). The highest median antibody level was observed in the 18–29 years group (316.27 U/mL, IQR: 212.71–334.49), whereas the lowest was recorded in participants aged ≥ 70 years (117.97 U/mL, IQR: 0–318.48). Ancestral RBD-blocking antibody levels also varied significantly by education level, comorbidities, and alcohol consumption (all *p* < 0.05). No significant differences were observed by sex, BMI, allergies, smoking status, or total physical activity per week (*p* > 0.05 for all comparisons, Table 3).

Participants with confirmed reinfection had higher ancestral RBD-blocking antibody levels (median: 305.69 U/mL, IQR: 74.51–335.41) than those without reinfection (253.72 U/mL, IQR: 36.33–330.60) and those with probable reinfection (176.86 U/mL, IQR: 0–327.59) (*p* = 0.001). Additionally, ancestral RBD-blocking antibody levels differed significantly by vaccination status (*p* < 0.001), with the highest antibody levels in heterologous booster vaccinated individuals (332.43 U/mL, IQR: 309.31–342.83) and the lowest antibody levels in unvaccinated individuals (0 U/mL, IQR: 0–37.58). No significant differences were observed by primary infection presentation or viral shedding of primary infection (*p* > 0.05 for all comparisons, Table 3).

To characterize the timing of immunologically relevant events relative to blood collection, we calculated the following intervals. Among reinfected participants (*n* = 465), the median time from reinfection to blood collection was 2.10 months (IQR: 1.64–2.86). Among non-reinfected participants who had received at least one vaccine dose (*n* = 1375), the median time from last immune event (the later of primary infection and last vaccine dose) to blood collection was 10.05 months (IQR: 9.26–10.48). Among non-reinfected participants who never received a vaccine (*n* = 254), the median time from primary infection to blood collection was 10.12 months (IQR: 9.77–10.81). These data are presented in Table A4.

### 3.5. Factors Associated with Ancestral RBD-Blocking Antibody Levels

A Tobit regression with bootstrap standard errors was performed to identify factors associated with ancestral RBD-blocking antibody levels (Table 4). The results showed that older age was associated with lower ancestral RBD-blocking antibody levels (β = –0.0038 per year, 95% bootstrap CI: –0.0057 to –0.0019, *p* < 0.001). Female sex was associated with higher ancestral RBD-blocking antibody levels than male sex (β = 0.0557, 95% CI: 0.0011 to 0.1082, *p* = 0.0478). Probable reinfection was associated with lower ancestral RBD-blocking antibody levels compared with non-reinfection (β = –0.3047, 95% CI: –0.4735 to –0.1446, *p* < 0.001). However, this finding may be influenced by the small number of probable cases (n = 108) and potential misclassification due to the symptom-based definition.

All vaccinated groups had significantly higher ancestral RBD-blocking antibody levels than unvaccinated individuals: partially vaccinated (β = 0.4440, 95% CI: 0.1569 to 0.6830, *p* < 0.001), fully vaccinated (β = 0.8516, 95% CI: 0.7464 to 0.9595, *p* < 0.001), homologous booster (β = 1.0297, 95% CI: 0.9408 to 1.1223, *p* < 0.001), and heterologous booster (β = 1.0838, 95% CI: 0.9387 to 1.2226, *p* < 0.001). Time since last immune event was inversely associated with ancestral RBD-blocking antibody levels (β = –0.0232 per month, 95% CI: –0.0385 to –0.0077, *p* = 0.0031).

A sensitivity analysis using quantile regression (Table A5) confirmed the robustness of these findings. The direction and significance of the main predictors (age, probable reinfection, vaccination status, and time since last immune event) were consistent with the primary model. In the quantile regression, partially vaccinated was not significant, and senior high school was associated with lower ancestral RBD-blocking antibody levels than junior high school or below (β = –0.0165, *p* = 0.0048), but this finding was not confirmed in the primary Tobit model (*p* = 0.0857) and requires replication in larger studies.

## 4. Discussion

In this cross-sectional study of 2095 individuals with prior Omicron BA.2 infection in Shanghai, we observed a high SARS-CoV-2 reinfection proportion of 21.8% (after age-sex standardization) during the early post-zero-COVID period (December 2022–April 2023). Meeting WHO physical activity recommendations (≥600 MET-min/week) was associated with lower reinfection odds (OR = 0.59, 95% CI: 0.46–0.74, *p* < 0.001). Regarding humoral immunity, the overall median ancestral RBD-blocking antibody level was 263.93 U/mL (IQR: 36.41–331.87). Older age was associated with lower ancestral RBD-blocking antibody levels (β = –0.0038 per year, 95% bootstrap CI: –0.0057 to –0.0019, *p* < 0.001). All vaccinated groups had significantly higher ancestral RBD-blocking antibody levels than unvaccinated individuals: partially vaccinated (β = 0.4440, 95% CI: 0.1569 to 0.6830, *p* < 0.001), fully vaccinated (β = 0.8516, 95% CI: 0.7464 to 0.9595, *p* < 0.001), homologous booster (β = 1.0297, 95% CI: 0.9408 to 1.1223, *p* < 0.001), and heterologous booster (β = 1.0838, 95% CI: 0.9387 to 1.2226, *p* < 0.001). Time since last immune event was inversely associated with ancestral RBD-blocking antibody levels (β = –0.0232 per month, 95% CI: –0.0385 to –0.0077, *p* = 0.0031).

SARS-CoV-2 reinfections increased substantially after the emergence of Omicron variants [4,5,6,21]. A UK study based on national COVID-19 Infection Survey data [21] reported that reinfection rates increased from 10–11% during the BA.1/BA.2 wave to 14–16% during the subsequent BA.4/BA.5 and later subvariants period. Following China’s policy adjustment in December 2022, a study in Guangdong province reported reinfection rates between December 2022 and January 2023 of 50.0% for wild-type strain, 35.2% for Alpha or Delta, and 18.4% for Omicron [18]. In the present study, the reinfection proportion was slightly higher than the domestic estimates. These differences may be attributed to variations in population immune background, survey periods, and sample size.

Notably, meeting the WHO-recommended physical activity level was associated with lower reinfection odds in our study. A possible mechanism for this association could involve improved mitochondrial metabolism and reduced levels of pro-inflammatory cytokines (e.g., IL-6), which may influence the immune microenvironment [26]. Previous studies have reported that moderate physical activity was associated with a lower risk of upper respiratory tract infections [27], and a Brazilian study reported that sufficient physical activity was associated with a lower risk of COVID-19-related hospitalization [28]. However, the cross-sectional design of our study does not allow us to determine temporality or causality. Reverse causation is a plausible alternative explanation: participants with better underlying health or fewer post-COVID symptoms may have been more likely to meet the WHO physical activity recommendations and also may have had lower reinfection risk. Therefore, these observations should be interpreted as associations only. Prospective studies are needed to further examine the relationship between physical activity and reinfection risk.

Our serological results showed that older age and longer time since the last immune event were associated with lower ancestral RBD-blocking antibody levels, whereas all vaccinated groups had higher ancestral RBD-blocking antibody levels than unvaccinated individuals. The observed inverse association between older age and RBD-blocking antibody levels is in line with previous studies identifying older age as an independent risk factor for impaired antibody responses [29,30], and likely reflects age-related immune senescence [31]. Previous research has shown that age-associated declines in T-cell help and germinal center reactions may contribute to diminished humoral immune responses after vaccination [32]. Similarly, the finding that longer time since the last immune event was associated with lower antibody levels is compatible with the known kinetics of antibody waning. Longitudinal studies have consistently shown that post-immunization antibody titers decrease over time [33,34]. Importantly, despite the negative associations with age and time since the last immune event, all vaccinated groups in our study exhibited higher ancestral RBD-blocking antibody levels than unvaccinated individuals. However, the cPass kit used in this study is based on the wild-type RBD. Therefore, the higher antibody levels observed in vaccinated groups reflect ancestral RBD-blocking activity and cannot be directly interpreted as protective against Omicron subvariants.

This study has several limitations. First, the cross-sectional design precludes causal inference. Therefore, all findings should be interpreted as associations rather than causal effects. Second, selection bias may exist because participants were slightly older than non-participants. Although age was adjusted for in the multivariable models and was not independently associated with reinfection, residual confounding by other unmeasured factors (e.g., disease severity, testing behavior, occupation, household size, mask use) cannot be excluded. Third, probable reinfections (23.2%) were defined based on symptoms and exposure history, which may be subject to misclassification. Sensitivity analyses restricted to confirmed reinfections showed consistent results for the association with physical activity. However, the associations for symptomatic primary infection and heterologous booster vaccination were no longer statistically significant. The estimate for heterologous booster vaccination was based on a very small number of events. The association between symptomatic primary infection and reinfection odds was not robust, which may reflect residual confounding, recall bias, or outcome misclassification. Consequently, both associations should be interpreted cautiously and require confirmation in larger studies. Fourth, the variable “vaccination after primary infection” should be interpreted cautiously because it occurred after the primary infection and may reflect post-infection health behavior, vaccination decisions, or other unmeasured factors rather than a simple baseline covariate. Fifth, the comparison of clinical symptoms between primary infection and reinfection is subject to surveillance bias. During primary infection (March–June 2022), routine nucleic acid amplification testing enabled detection of asymptomatic cases. After December 2022, following the adjustment of the zero-COVID policy, large-scale testing was largely discontinued. This differential case ascertainment inevitably leads to a higher reported symptom prevalence during reinfection, regardless of true clinical severity. Hence, our symptom comparisons cannot provide reliable evidence that reinfection is biologically more symptomatic than primary infection. Similarly, measurements of viral shedding duration during the zero-COVID period may also be influenced by differential testing access and frequency. Therefore, any such associations should be interpreted with caution. Sixth, the cPass assay used in this study is based on the wild-type RBD. Therefore, the measured levels primarily reflect antibody-mediated blocking of the ancestral SARS-CoV-2 RBD–ACE2 interaction and may not fully capture functional neutralization, particularly against Omicron subvariants. In our study, antibody levels were used as a quantitative measure of humoral immune response and were not intended as a direct correlate of protection against Omicron reinfection. Seventh, because antibody levels were measured after the reinfection observation period, reinfection status may reflect post-reinfection immune boosting; therefore, the antibody model should not be interpreted as estimating only baseline predictors of pre-reinfection antibody levels. Eighth, this study was conducted in one district of Shanghai during two specific periods when Omicron BA.2 and BA.5 were the dominant subvariants, respectively. The findings may not be generalizable to other geographical regions, to later Omicron subvariants, or to populations with different immune backgrounds. Finally, the sample size in the aged <18 years group was relatively small, limiting subgroup analyses. Future prospective cohort studies incorporating asymptomatic screening, variant-covering serological assays, and longitudinal follow-up are needed to extend our understanding of SARS-CoV-2 reinfection and long-term immune responses.

## 5. Conclusions

This study revealed that Omicron reinfection was common (21.8%) during the early post-zero-COVID period in Shanghai. Meeting WHO physical activity recommendations was associated with 41% lower reinfection odds, although reverse causality cannot be excluded. All vaccinated groups showed higher ancestral RBD-blocking antibody levels than unvaccinated individuals, with the highest median levels observed among heterologous booster recipients; however, this subgroup was small and the assay measured ancestral RBD–ACE2 blocking rather than Omicron-specific neutralization. Older age and longer time since the last immune event were associated with lower ancestral RBD-blocking antibody levels. Due to the cross-sectional design, causal inferences are not supported and the antibody assay does not directly measure Omicron-specific neutralization. Future prospective studies with well-powered designs and variant-specific assays are needed to further examine these associations.

## Figures and Tables

**Table 1:** Baseline characteristics of participants by SARS-CoV-2 reinfection status.

	Total (*n* = 2095)	Non-Reinfection (*n* = 1630)	Reinfection			*p* *
			Total (*n* = 465)	Confirmed (*n* = 357)	Probable (*n* = 108)	
Age, yr						
Median (IQR)	56 (42–68)	56 (42–68)	57 (42–68)	57 (42–68)	57.5 (43–68)	0.914
<18, n (%)	32 (1.53)	25 (1.53)	7 (1.51)	4 (1.12)	3 (2.78)	0.241
18–29, n (%)	126 (6.01)	104 (6.38)	22 (4.73)	19 (5.32)	3 (2.78)	
30–39, n (%)	273 (13.03)	205 (12.58)	68 (14.62)	54 (15.13)	14 (12.96)	
40–49, n (%)	343 (16.37)	266 (16.32)	77 (16.56)	59 (16.53)	18 (16.67)	
50–59, n (%)	399 (19.05)	321 (19.69)	78 (16.77)	60 (16.81)	18 (16.67)	
60–69, n (%)	515 (24.58)	385 (23.62)	130 (27.96)	102 (28.57)	28 (25.93)	
≥70, n (%)	407 (19.43)	324 (19.88)	83 (17.85)	59 (16.53)	24 (22.22)	
Sex						
Male	963 (45.97)	761 (46.69)	202 (43.44)	145 (40.62)	57 (52.78)	0.215
Female	1132 (54.03)	869 (53.31)	263 (56.56)	212 (59.38)	51 (47.22)	
BMI						
<18.5	75 (3.58)	66 (4.05)	9 (1.94)	8 (2.24)	1 (0.93)	0.105
18.5–23.9	1000 (47.73)	763 (46.81)	237 (50.97)	180 (50.42)	57 (52.78)	
24–27.9	760 (36.28)	597 (36.63)	163 (35.05)	122 (34.17)	41 (37.96)	
≥28	260 (12.41)	204 (12.51)	56 (12.04)	47 (13.17)	9 (8.33)	
Education level						
Junior high school or below	835 (39.86)	645 (39.57)	190 (40.86)	139 (38.94)	51 (47.22)	0.435
Senior high school	577 (27.54)	460 (28.22)	117 (25.16)	86 (24.09)	31 (28.70)	
College or bachelor’s degree	611 (29.16)	473 (29.02)	138 (29.68)	115 (32.21)	23 (21.30)	
Graduate degree or higher	72 (3.44)	52 (3.19)	20 (4.30)	17 (4.76)	3 (2.78)	
Comorbidities						
Yes	772 (36.85)	607 (37.24)	165 (35.48)	130 (36.41)	35 (32.41)	0.489
No	1323 (63.15)	1023 (62.76)	300 (64.52)	227 (63.59)	73 (67.59)	
Allergies						
Yes	192 (9.16)	158 (9.69)	34 (7.31)	28 (7.84)	6 (5.56)	0.116
No	1903 (90.84)	1472 (90.31)	431 (92.69)	329 (92.16)	102 (94.44)	
Smoking status						
Current smoker	227 (10.84)	170 (10.43)	57 (12.26)	46 (12.89)	11 (10.19)	0.247
Former smoker	75 (3.58)	63 (3.87)	12 (2.58)	8 (2.24)	4 (3.7)	
Never smoker	1793 (85.58)	1397 (85.71)	396 (85.16)	303 (84.87)	93 (86.11)	
Alcohol consumption						
Current drinker	246 (11.74)	198 (12.15)	48 (10.32)	40 (11.21)	8 (7.41)	0.431
Former drinker	119 (5.68)	89 (5.46)	30 (6.45)	20 (5.60)	10 (9.26)	
Never drinker	1730 (82.58)	1343 (82.39)	387 (83.23)	297 (83.19)	90 (83.33)	
Total physical activity per week						
Meeting WHO recommendations	790 (37.71)	653 (40.06)	137 (29.46)	108 (30.25)	29 (26.85)	<0.001
Not meeting WHO recommendations	1305 (62.29)	977 (59.94)	328 (70.54)	249 (69.75)	79 (73.15)	
Primary infection presentation						
Asymptomatic	833 (39.76)	669 (41.04)	164 (35.27)	131 (36.69)	33 (30.56)	0.025
Symptomatic	1262 (60.24)	961 (58.96)	301 (64.73)	226 (63.31)	75 (69.44)	
Viral shedding of primary infection ^&^						
≤14 days	1805 (86.16)	1390 (85.28)	415 (89.25)	314 (87.96)	101 (93.52)	0.029
>14 days	290 (13.84)	240 (14.72)	50 (10.75)	43 (12.04)	7 (6.48)	
Vaccination status ^#^						
Unvaccinated	329 (15.71)	254 (15.59)	75 (16.13)	57 (15.97)	18 (16.67)	0.013
Partially vaccinated	38 (1.81)	28 (1.72)	10 (2.15)	8 (2.24)	2 (1.85)	
Fully vaccinated	419 (20.01)	328 (20.14)	91 (19.57)	77 (21.57)	14 (12.96)	
Homologous booster vaccinated	1222 (58.36)	939 (57.64)	283 (60.86)	210 (58.82)	73 (67.59)	
Heterologous booster vaccinated	86 (4.11)	80 (4.91)	6 (1.29)	5 (1.40)	1 (0.93)	
Vaccination after primary infection ^#^						
Yes	181 (8.64)	156 (9.58)	25 (5.38)	20 (5.60)	5 (5.63)	0.004
No	1913 (91.36)	1473 (90.42)	440 (94.62)	337 (94.40)	103 (95.37)	

* *p* value for comparison between non-reinfection and total reinfection groups. No adjustment for multiple testing was applied, as the analyses were primarily exploratory. *p*-values should be interpreted descriptively. ^&^ Viral shedding duration was defined as the time interval from the first positive SARS-CoV-2 RNA test to the first negative SARS-CoV-2 RNA test result. ^#^ One case (0.05%) with missing vaccination data was excluded.

**Table 2:** Univariable and multivariable logistic regression analyses for SARS-CoV-2 reinfection *.

Factors	Univariable Analysis		*p*	Multivariable Analysis		*p*
	OR	95% CI		OR	95% CI	
Age, yr						
<18	Ref			Ref		
18–29	0.76	(0.29–1.97)	0.565	0.62	(0.22–1.70)	0.351
30–39	1.19	(0.49–2.86)	0.706	0.91	(0.35–2.36)	0.838
40–49	1.03	(0.43–2.48)	0.941	0.81	(0.31–2.07)	0.652
50–59	0.87	(0.36–2.08)	0.751	0.69	(0.27–1.77)	0.440
60–69	1.21	(0.51–2.85)	0.670	0.98	(0.39–2.50)	0.972
≥70	0.91	(0.38–2.19)	0.842	0.74	(0.29–1.92)	0.535
Sex						
Male	Ref			Ref		
Female	1.14	(0.93–1.40)	0.216	1.13	(0.89–1.44)	0.321
BMI						
≥28	Ref			Ref		
<18.5	0.50	(0.23–1.06)	0.070	0.45	(0.21–1.01)	0.050
18.5–23.9	1.13	(0.81–1.57)	0.463	1.09	(0.78–1.54)	0.614
24–27.9	0.99	(0.71–1.40)	0.975	1.03	(0.72–1.46)	0.878
Education level						
Junior high school or below	Ref			Ref		
Senior high school	0.86	(0.67–1.12)	0.268	0.81	(0.62–1.06)	0.126
College or bachelor’s degree	0.99	(0.77–1.27)	0.940	1.04	(0.77–1.39)	0.819
Graduate degree or higher	1.31	(0.76–2.24)	0.333	1.50	(0.82–2.72)	0.188
Comorbidities						
No	Ref			Ref		
Yes	0.93	(0.75–1.15)	0.489	0.89	(0.69–1.15)	0.381
Smoking status						
Never smoker	Ref			Ref		
Current smoker	1.18	(0.86–1.63)	0.304	1.39	(0.94–2.06)	0.095
Former smoker	0.67	(0.36–1.26)	0.214	0.88	(0.45–1.72)	0.711
Alcohol consumption						
Never drinker	Ref			Ref		
Current drinker	0.84	(0.60–1.18)	0.312	0.83	(0.56–1.23)	0.357
Former drinker	1.17	(0.76–1.80)	0.474	1.10	(0.69–1.77)	0.685
Total physical activity per week						
Not meeting WHO recommendations	Ref			Ref		
Meeting WHO recommendations	0.62	(0.50–0.78)	<0.001	0.59	(0.46–0.74)	<0.001
Primary infection presentation						
Asymptomatic	Ref			Ref		
Symptomatic	1.28	(1.03–1.58)	0.025	1.37	(1.09–1.72)	0.008
Viral shedding of primary infection						
≤14 days	Ref			Ref		
>14 days	0.70	(0.50–0.96)	0.029	0.73	(0.52–1.01)	0.057
Vaccination status						
Unvaccinated	Ref			Ref		
Partially vaccinated	1.21	(0.56–2.60)	0.627	1.35	(0.60–3.08)	0.471
Fully vaccinated	0.94	(0.66–1.33)	0.725	0.96	(0.67–1.40)	0.846
Homologous booster vaccinated	1.02	(0.76–1.36)	0.890	1.06	(0.77–1.44)	0.732
Heterologous booster vaccinated	0.25	(0.11–0.61)	0.002	0.31	(0.11–0.89)	0.029
Vaccination after primary infection						
No	Ref			Ref		
Yes	0.54	(0.35–0.83)	0.005	0.83	(0.48–1.43)	0.505

* One case (0.05%) with missing vaccination data was excluded. OR, odds ratio; CI, confidence interval. No adjustment for multiple testing was applied, as the analyses were primarily exploratory. *p*-values should be interpreted descriptively.

**Table 3:** Ancestral RBD-blocking antibody levels by participant characteristics.

	*n*	Median (IQR)	*p*
Overall	2095	263.93 (36.41–331.87)	-
Age, yr			
<18	32	274.96 (94.73–338.49)	<0.001
18–29	126	316.27 (212.71–334.49)	
30–39	273	306.59 (99.53–335.87)	
40–49	343	300.35 (99.52–331.87)	
50–59	399	293.03 (66.08–335.76)	
60–69	515	209.51 (0–326.71)	
≥70	407	117.97 (0–318.48)	
Sex			
Male	963	246.56 (39.53–328.42)	0.241
Female	1132	274.18 (33.46–333.37)	
BMI			
<18.5	75	266.42 (36.33–326.71)	0.752
18.5–24	1274	260.97 (36.83–332.38)	
25–28	487	273.27 (40.27–331.03)	
>28	259	263.04 (26.21–328.42)	
Education level			
Junior high school or below	835	251.72 (28.04–330.14)	<0.001
Senior high school	577	214.36 (31.53–326.19)	
College or bachelor’s degree	611	303.23 (73.41–335.41)	
Graduate degree or higher	72	311.42 (78.62–339.92)	
Comorbidities			
Yes	772	159.94 (0–325.03)	<0.001
No	1323	294.23 (79.74–333.63)	
Allergies			
Yes	192	232.40 (29.42–331.61)	0.632
No	1903	265.31 (37.08–331.87)	
Smoking status			
Current smoker	227	259.89 (46.40–328.96)	0.846
Former smoker	75	232.01 (0–332.99)	
Never smoker	1793	264.21 (36.02–331.87)	
Alcohol consumption			
Current drinker	246	279.96 (58.90–335.46)	0.014
Former drinker	119	301.67 (107.78–337.85)	
Never drinker	1730	260.41 (32.25–330.26)	
Total physical activity per week			
Meeting WHO recommendations	790	276.21 (62.98–331.87)	0.075
Not meeting WHO recommendations	1305	253.64 (29.57–331.41)	
Primary infection presentation			
Asymptomatic	833	259.05 (37.08–328.42)	0.115
Symptomatic	1262	267.20 (36.22–333.37)	
Viral shedding of primary infection			
≤14 days	1805	266.31 (37.03–331.87)	0.412
>14 days	290	224.27 (35.41–330.84)	
Reinfection status			
Non-reinfection	1630	253.72 (36.33–330.60)	0.001
Reinfection (Confirmed cases)	357	305.69 (74.51–335.41)	
Reinfection (Probable cases)	108	176.86 (0–327.59)	
Vaccination status *			
Unvaccinated	327	0 (0–37.58)	<0.001
Partially vaccinated	39	39.30 (0–172.60)	
Fully vaccinated	417	239.45 (56.93–328.42)	
Homologous booster vaccinated	1209	306.59 (125.74–335.41)	
Heterologous booster vaccinated	102	332.43 (309.31–342.83)	

* One case (0.05%) with missing vaccination data was excluded. No adjustment for multiple testing was applied, as the analyses were primarily exploratory. *p*-values should be interpreted descriptively.

**Table 4:** Tobit regression (bootstrap standard errors) for factors associated with ancestral RBD-blocking antibody levels.

	Coefficient	Bootstrap SE	Bootstrap 95% CI	*p* *
Age, yr	−0.0038	0.0010	(−0.0057 to −0.0019)	<0.001
Sex				
Male	Ref			
Female	0.0557	0.0281	(0.0011 to 0.1082)	0.0478
BMI	−0.0010	0.0035	(−0.0075 to 0.0060)	0.7611
Education level				
Junior high school or below	Ref			
Senior high school	−0.0525	0.0306	(−0.1114 to 0.0052)	0.0857
College or bachelor’s degree	−0.0366	0.0317	(−0.1040 to 0.0228)	0.2480
Graduate degree or higher	−0.0932	0.0743	(−0.2477 to 0.0386)	0.2094
Comorbidities				
No	Ref			
Yes	−0.0578	0.0300	(−0.1206 to −0.0008)	0.0543
Smoking status				
Never smoker	Ref			
Current smoker	−0.0426	0.0477	(−0.1383 to 0.0467)	0.3715
Former smoker	0.0633	0.0714	(−0.0794 to 0.2017)	0.3754
Alcohol consumption				
Never drinker	Ref			
Current drinker	−0.0210	0.0470	(−0.1129 to 0.0709)	0.6557
Former drinker	0.0609	0.0520	(−0.0428 to 0.1608)	0.2418
Total physical activity per week				
Not meeting WHO recommendations	Ref			
Meeting WHO recommendations	−0.0097	0.0256	(−0.0597 to 0.0383)	0.7046
Reinfection status				
Non-reinfection	Ref			
Reinfection (Confirmed cases)	−0.0775	0.0632	(−0.2056 to 0.0391)	0.2198
Reinfection (Probable cases)	−0.3047	0.0869	(−0.4735 to −0.1446)	<0.001
Vaccination status ^#^				
Unvaccinated	Ref			
Partially vaccinated	0.4440	0.1348	(0.1569 to 0.6830)	<0.001
Fully vaccinated	0.8516	0.0543	(0.7464 to 0.9595)	<0.001
Homologous booster vaccinated	1.0297	0.0468	(0.9408 to 1.1223)	<0.001
Heterologous booster vaccinated	1.0838	0.0723	(0.9387 to 1.2226)	<0.001
Time since last immune event, months	−0.0232	0.0078	(−0.0385 to −0.0077)	0.0031

^#^ One case (0.05%) with missing vaccination data was excluded. * *p* values based on bootstrap standard errors using normal approximation. Bootstrap based on 1000 resamples. No adjustment for multiple testing was applied, as the analyses were primarily exploratory. *p*-values should be interpreted descriptively.

## Data Availability

The datasets generated during the current study are available from the corresponding author upon reasonable request.

## Appendix A

**Table A1:** Comparison of demographic characteristics between participants and non-participants.

	Total (*n* = 3863)	Participants (*n* = 2095)	Non-Participants (*n* = 1768)	*p*
Age, yr				
Median (IQR)	54 (39–67)	56 (42–68)	52 (33–66)	<0.001
<18, n (%)	151 (3.91)	32 (1.53)	119 (6.73)	<0.001
18–29, n (%)	345 (8.93)	126 (6.01)	219 (12.39)	
30–39, n (%)	508 (13.15)	273 (13.03)	235 (13.29)	
40–49, n (%)	558 (14.44)	343 (16.37)	215 (12.16)	
50–59, n (%)	686 (17.76)	399 (19.05)	287 (16.23)	
60–69, n (%)	880 (22.78)	515 (24.58)	365 (20.65)	
≥70, n (%)	735 (19.03)	407 (19.43)	328 (18.55)	
Sex				
Male	1789 (46.31)	963 (45.97)	826 (46.72)	0.650
Female	2074 (53.69)	1132 (54.03)	942 (53.28)	

**Table A2:** Sensitivity analysis of factors associated with SARS-CoV-2 reinfection restricted to confirmed cases (n = 357) *.

Factors	Univariable Analysis		*p*	Multivariable Analysis		*p*
	OR	95% CI		OR	95% CI	
Age, yr						
<18	Ref			Ref		
18–29	1.14	(0.36–3.65)	0.823	0.88	(0.26–3.01)	0.844
30–39	1.65	(0.55–4.93)	0.373	1.18	(0.37–3.81)	0.779
40–49	1.39	(0.47–4.13)	0.558	1.01	(0.32–3.24)	0.981
50–59	1.17	(0.39–3.48)	0.780	0.92	(0.29–2.92)	0.886
60–69	1.66	(0.56–4.87)	0.359	1.32	(0.42–4.18)	0.632
≥70	1.14	(0.38–3.39)	0.816	0.90	(0.28–2.9)	0.859
Sex						
Male	Ref			Ref		
Female	1.28	(1.02–1.62)	0.037	1.34	(1.02–1.75)	0.038
BMI						
≥28	Ref			Ref		
<18.5	0.53	(0.24–1.17)	0.115	0.48	(0.21–1.10)	0.081
18.5–23.9	1.02	(0.72–1.46)	0.896	0.96	(0.67–1.39)	0.843
24–27.9	0.89	(0.61–1.29)	0.528	0.91	(0.63–1.34)	0.644
Education level						
Junior high school or below	Ref			Ref		
Senior high school	0.87	(0.65–1.16)	0.344	0.80	(0.59–1.08)	0.148
College or bachelor’s degree	1.13	(0.86–1.48)	0.388	1.21	(0.88–1.67)	0.247
Graduate degree or higher	1.52	(0.85–2.70)	0.157	1.82	(0.96–3.46)	0.066
Comorbidities						
No	Ref			Ref		
Yes	0.97	(0.76–1.22)	0.770	0.96	(0.73–1.28)	0.800
Smoking status						
Never smoker	Ref			Ref		
Current smoker	1.25	(0.88–1.77)	0.214	1.62	(1.06–2.47)	0.026
Former smoker	0.59	(0.28–1.24)	0.160	0.85	(0.38–1.87)	0.683
Alcohol consumption						
Never drinker	Ref			Ref		
Current drinker	0.91	(0.64–1.31)	0.625	0.86	(0.56–1.32)	0.491
Former drinker	1.02	(0.62–1.68)	0.950	0.93	(0.54–1.61)	0.795
Total physical activity per week						
Not meeting WHO recommendations	Ref			Ref		
Meeting WHO recommendations	0.65	(0.51–0.83)	0.001	0.61	(0.47–0.79)	<0.001
Primary infection presentation						
Asymptomatic	Ref			Ref		
Symptomatic	1.20	(0.95–1.52)	0.130	1.22	(0.95–1.57)	0.127
Viral shedding of primary infection						
≤14 days	Ref			Ref		
>14 days	0.79	(0.56–1.12)	0.190	0.82	(0.58–1.17)	0.283
Vaccination status						
Unvaccinated	Ref			Ref		
Partially vaccinated	1.27	(0.55–2.94)	0.572	1.46	(0.59–3.59)	0.410
Fully vaccinated	1.05	(0.72–1.53)	0.816	1.09	(0.73–1.64)	0.663
Homologous booster vaccinated	1.00	(0.72–1.38)	0.983	1.04	(0.74–1.47)	0.828
Heterologous booster vaccinated	0.28	(0.11–0.72)	0.008	0.32	(0.10–1.01)	0.052
Vaccination after primary infection						
No	Ref			Ref		
Yes	0.56	(0.35–0.91)	0.018	0.87	(0.47–1.59)	0.650

* One case (0.05%) with missing vaccination data was excluded. No adjustment for multiple testing was applied, as the analyses were primarily exploratory. *p*-values should be interpreted descriptively.

**Table A3:** Comparison of clinical symptoms between primary infection and reinfection among the 465 reinfection cases.

	Primary Infection (*n* = 465)	Reinfection (*n* = 465)	*p* *
Clinical manifestation, n (%)			
Asymptomatic	164 (35.27)	33 (7.10)	<0.001
Symptomatic	301 (64.73)	432 (92.90)	
Specific symptoms, n (%)			
Fever	217 (46.67)	294 (63.23)	<0.001
High fever (>38 °C)	179 (38.49)	226 (48.60)	0.001
Cough	124 (26.67)	209 (44.95)	<0.001
Sore throat	100 (21.51)	232 (49.89)	<0.001
Fatigue	86 (18.49)	179 (38.49)	<0.001
Nasal congestion	37 (7.96)	97 (20.86)	<0.001
Expectoration	30 (6.45)	79 (16.99)	<0.001
Headache	29 (6.24)	60 (12.90)	<0.001
Runny nose	28 (6.02)	94 (20.22)	<0.001
Myalgia	26 (5.59)	73 (15.70)	<0.001
Loss of appetite	18 (3.87)	45 (9.68)	<0.001
Anosmia/ageusia	17 (3.66)	38 (8.17)	0.001
Chest tightness	15 (3.23)	30 (6.45)	0.008
Chill	13 (2.80)	49 (10.54)	<0.001
Dizziness	11 (2.37)	42 (9.03)	<0.001
Diarrhea	8 (1.72)	13 (2.80)	0.267
Vomiting	8 (1.72)	5 (1.08)	0.453
shortness of breath	5 (1.08)	9 (1.94)	0.344
Nausea	5 (1.08)	7 (1.51)	0.727
Other symptoms	2 (0.43)	7 (1.51)	0.180
Conjunctival congestion	1 (0.22)	2 (0.43)	1.000
Limb edema	0 (0)	2 (0.43)	-

* McNemar’s test for paired comparisons. No adjustment for multiple testing was applied, as the analyses were primarily exploratory. *p*-values should be interpreted descriptively. Symptom comparisons are confounded by surveillance bias: primary infection was detected under routine nucleic acid amplification testing, while reinfections were largely symptom-triggered after testing was discontinued.

**Table A4:** Time since last immune event to blood collection, stratified by reinfection and vaccination status.

Subgroup	*n* *	Median (IQR), Months
Reinfected participants	465	2.10 (1.64–2.86)
Non-reinfected, vaccinated	1375	10.05 (9.26–10.48)
Non-reinfected, unvaccinated	254	10.12 (9.77–10.81)

* One case (0.05%) with missing vaccination data was excluded. Time since last immune event was defined as the interval between blood collection and the latest of: primary infection date, last vaccine dose (if any), or reinfection date (if any). For non-reinfected vaccinated participants, the most recent event was therefore the later of primary infection date and last vaccine dose.

**Table A5:** Quantile regression (τ = 0.5) for factors associated with ancestral RBD-blocking antibody levels.

	Coefficient	SE	*p*
Age, yr	−0.0008	0.0003	0.0020
Sex			
Male	Ref		
Female	0.0101	0.0059	0.0889
BMI	−0.0001	0.0005	0.7794
Education level			
Junior high school or below	Ref		
Senior high school	−0.0165	0.0058	0.0048
College or bachelor’s degree	−0.0007	0.0058	0.9019
Graduate degree or higher	−0.0219	0.0128	0.0884
Comorbidities			
No	Ref		
Yes	0.0001	0.0054	0.9885
Smoking status			
Never smoker	Ref		
Current smoker	−0.0100	0.0155	0.5190
Former smoker	0.0104	0.0104	0.3190
Alcohol consumption			
Never drinker	Ref		
Current drinker	−0.0055	0.0096	0.5658
Former drinker	0.0106	0.0148	0.4709
Total physical activity per week			
Not meeting WHO recommendations	Ref		
Meeting WHO recommendations	−0.0013	0.0045	0.7738
Reinfection status			
Non-reinfection	Ref		
Reinfection (Confirmed cases)	0.0018	0.0060	0.7680
Reinfection (Probable cases)	−0.0450	0.0174	0.0098
Vaccination status			
Unvaccinated	Ref		
Partially vaccinated	0.3421	0.4130	0.4076
Fully vaccinated	1.1373	0.0332	<0.001
Homologous booster vaccinated	1.2433	0.0088	<0.001
Heterologous booster vaccinated	1.2488	0.0129	<0.001
Time since last immune event, months	−0.0032	0.0011	0.0042

No adjustment for multiple testing was applied, as the analyses were primarily exploratory. *p*-values should be interpreted descriptively.
